# Attenuated cerebrospinal fluid leukocyte count and sepsis in adults with pneumococcal meningitis: a prospective cohort study

**DOI:** 10.1186/1471-2334-6-149

**Published:** 2006-10-12

**Authors:** Martijn Weisfelt, Diederik van de Beek, Lodewijk Spanjaard, Johannes B Reitsma, Jan de Gans

**Affiliations:** 1 Department of Neurology, Centre of Infection and Immunity Amsterdam (CINIMA), Academic Medical Centre, University of Amsterdam, Amsterdam, The Netherlands; 2 Department of Medical Microbiology/Netherlands Reference Laboratory of Bacterial Meningitis, Centre of Infection and Immunity Amsterdam (CINIMA), Academic Medical Centre, University of Amsterdam, Amsterdam, The Netherlands; 3 Department of Clinical Epidemiology and Biostatistics, Centre of Infection and Immunity Amsterdam (CINIMA), Academic Medical Centre, University of Amsterdam, Amsterdam, The Netherlands

## Abstract

**Background:**

A low cerebrospinal fluid (CSF) white-blood cell count (WBC) has been identified as an independent risk factor for adverse outcome in adults with bacterial meningitis. Whereas a low CSF WBC indicates the presence of sepsis with early meningitis in patients with meningococcal infections, the relation between CSF WBC and outcome in patients with pneumococcal meningitis is not understood.

**Methods:**

We examined the relation between CSF WBC, bacteraemia and sepsis in a prospective cohort study that included 352 episodes of pneumococcal meningitis, confirmed by CSF culture, occurring in patients aged >16 years.

**Results:**

CSF WBC was recorded in 320 of 352 episodes (91%). Median CSF WBC was 2530 per mm^3 ^(interquartile range 531–6983 per mm^3^) and 104 patients (33%) had a CSF WBC <1000/mm^3^. Patients with a CSF WBC <1000/mm^3 ^were more likely to have an unfavourable outcome (defined as a Glasgow Outcome Scale score of 1–4) than those with a higher WBC (74 of 104 [71%] vs. 87 of 216 [43%]; *P *< 0.001). CSF WBC was significantly associated with blood WBC (Spearman's test 0.29), CSF protein level (0.20), thrombocyte count (0.21), erythrocyte sedimentation rate (-0.15), and C-reactive protein levels (-0.18). Patients with a CSF WBC <1000/mm^3 ^more often had a positive blood culture (72 of 84 [86%] vs. 138 of 196 [70%]; *P *= 0.01) and more often developed systemic complications (cardiorespiratory failure, sepsis) than those with a higher WBC (53 of 104 [51%] vs. 69 of 216 [32%]; *P *= 0.001). In a multivariate analysis, advanced age (Odds ratio per 10-year increments 1.22, 95%CI 1.02–1.45), a positive blood culture (Odds ratio 2.46, 95%CI 1.17–5.14), and a low thrombocyte count on admission (Odds ratio per 100,000/mm^3 ^increments 0.67, 95% CI 0.47–0.97) were associated with a CSF WBC <1000/mm^3^.

**Conclusion:**

A low CSF WBC in adults with pneumococcal meningitis is related to the presence of signs of sepsis and systemic complications. Invasive pneumococcal infections should possibly be regarded as a continuum from meningitis to sepsis.

## Background

Bacterial meningitis is a life-threatening disease [1,2]. Recently we evaluated clinical features and prognostic factors in a prospective cohort study of 696 episodes of community-acquired acute bacterial meningitis [3]. The most common pathogens were *Streptococcus pneumoniae *and *Neisseria meningitidis*. The mortality rate was 21 percent and was higher among patients with pneumococcal meningitis than among those with meningococcal meningitis (30% vs. 7%, *P *< 0.001). The outcome was unfavourable in 34 percent of episodes and the strongest risk factors for an unfavourable outcome were symptoms indicative of systemic compromise, a low level of consciousness, infection with *S. pneumoniae*, and a low cerebrospinal fluid (CSF) white-blood cell count (WBC). The relation between unfavourable outcome and a low CSF WBC has been described previously in both patients with *N. meningitidis *and *S. pneumoniae *meningitis [3-6]. In patients with meningococcal meningitis, a low CSF WBC indicates the presence of sepsis with early meningitis [3]. The relation between CSF WBC and outcome in patients with pneumococcal meningitis is not understood [2-4]. A recent study in experimental pneumococcal meningitis suggested that an attenuated CSF pleocytosis was likely the result of a decrease in blood WBC induced by bacteraemia [7]. This suggests that a low CSF WBC in patients with pneumococcal meningitis may indicate the presence of sepsis, analogous to meningococcal disease. The aim of the present study was to examine the relation between CSF pleocytosis, bacteraemia and sepsis in a prospective cohort study of adults with pneumococcal meningitis.

## Methods

The Dutch Meningitis Cohort Study, a prospective nationwide observational cohort study in the Netherlands, includes 696 episodes of community-acquired bacterial meningitis [3]. Inclusion and exclusion criteria are described more extensively elsewhere [3]. In summary, eligible patients were aged >16 years and had CSF culture proven bacterial meningitis. CSF culture yielded *S. pneumoniae *in 352 episodes (51%), *N. meningitidis *in 257 episodes (37%), and other bacteria in 87 episodes (13%) [3]. The Dutch Meningitis Cohort Study was approved by our ethics committee and written informed consent was obtained from participating patients or their legally authorised representatives. Information was collected by means of a case record form. Patients using immunosuppressive drugs and patients with diabetes mellitus, alcoholism, asplenia or infection with the human immunodeficiency virus were considered immunocompromised. Fever was defined as a body temperature ≥38°C. A diastolic blood pressure <60 mm Hg was used as a surrogate marker for septic shock. Patients underwent a neurological examination at discharge and outcome was graded by means of the Glasgow Outcome Scale (GOS). This is a well validated measurement scale with scores varying from 1 (indicating death) to 5 (good recovery) [3,8]. A favourable outcome was defined as a score of 5, and an unfavourable outcome as a score 1–4.

For nonparametric testing, χ^2^- or Fisher's exact statistics were used. Correlation between parameters was performed by non-parametric Spearman rank test. We used logistic regression analysis to calculate odds ratios and 95% confidence intervals (CI) to assess the strength of the association between potential factors and a low CSF WBC (defined as a CSF WBC <1000/mm^3^). Although the median percentage of missing values for potential factors was low (2%), data were complete for all factors in only 175 episodes (50%) [4]. Missing values were imputed by use of multivariate normal distributions, and the coefficients of ten rounds of imputation were combined to obtain the final estimates from the multivariate model [3,4]. All statistical tests were two-tailed, and we considered a P value less than 0.05 as statistically significant. All analyses were undertaken with SAS software (version 9.11).

## Results

A total of 352 episodes of pneumococcal meningitis occurred in 343 patients; 9 patients had a second episode during the study period. Characteristics of the study population, clinical course and outcome are described more extensively elsewhere [3,4].

All patients underwent lumbar puncture. CSF WBC was determined in 320 episodes (91%); median CSF WBC was 2530 per mm^3 ^(interquartile range 531 to 6983 per mm^3^) and in 104 of 320 episodes (33%) CSF WBC was <1000/mm^3 ^(Table 1). Patients with a CSF WBC <1000/mm^3 ^were significantly older than those with a higher CSF WBC (mean age 62 vs. 57 years; *P *= 0.01), were less likely to have complaints <24 hours before admission (39 of 97 [40%] vs. 110 of 200 [55%]; *P *= 0.02), more often had a positive blood culture (72 of 84 [86%] vs. 138 of 196 [70%]; *P *= 0.01) and an immunocompromised status (31 of 104 [30%] vs. 39 of 215 [18%]; *P *= 0.02). Patients with a CSF WBC below or above 1000 per mm^3 ^had similar proportions of patients with fever, tachycardia (heart rate >120 beats/min) or diastolic blood pressure <60 mmHg on admission. Although several factors were significantly associated with a low CSF WBC in the univariate analysis, most factors were not significant in the multivariate analysis. In the multivariate model, advanced age (Odds ratio per 10-year increments 1.22, 95%CI 1.02–1.45), a positive blood culture (Odds ratio 2.46, 95%CI 1.17–5.14), and a low thrombocyte count on admission (Odds ratio per 100,000/mm^3 ^increments 0.67, 95%CI 0.47–0.97) were significantly associated with a CSF WBC <1000/mm^3^.

**Table 1:** Multivariate analysis of factors associated a low cerebrospinal fluid leukocyte count*

**Characteristics**	**CSF WBC <1000/mm^**3 **^(n = 104)**	**CSF WBC ≥1000/mm^**3 **^(n = 216)**	**Odds Ratio (95% CI)†**	**P-value**
Mean age ± SD – years	62 ± 17	57 ± 16	1.22 (1.02–1.45)	0.03‡
Duration of symptoms <24 hr	39/97 (40)	110/200 (55)	1.59 (0.93–2.72)	0.09‡
Immunocompromise§	31 (30)	39/215 (18)	1.74 (0.95–3.18)	0.07‡
Heart rate < 60 beats/min	3/101 (3)	3/202 (1)	3.90 (0.75–20.29)	0.11
Heart rate 60–90 beats/min	26/101 (26)	60/202 (30)	- ¶	
Heart rate 90–120 beats/min	45/101 (45)	91/202 (45)	1.00 (0.53–1.89)	0.99
Heart rate >120 beats/min	27/101 (27)	48/202 (24)	1.22(0.52–2.90)	0.65
Diastolic blood pressure <60 mmHg	5/103 (5)	12/209 (6)	0.79 (0.23–2.67)	0.70
Body temperature ≥38°C	84/103 (82)	181/212 (85)	0.59 (0.26–1.29)	0.18
Glasgow Coma Scale score$	11 (8–13)	10 (8–12)	1.08 (0.96–1.20)	0.20
Focal neurologic deficits	47 (45)	93 (43)	1.14 (0.67–1.94)	0.64
CSF protein level – g/L$	3.9 (2.2–6.3)	4.7 (2.9–7.1)	0.94 (0.85–1.03)	0.18
CSF:blood glucose ratio$	0.06 (0.01–0.17)	0.05 (0.01–0.23)	1.08 (0.79–1.49)	0.62
Positive blood culture	72/84 (86)	138/196 (70)	2.46 (1.17–5.14)	0.02‡
ESR – mm/hour$	47 (23–87)	43 (20–71)	1.14 (0.94–1.39)	0.18
Blood WBC <10 – 10^9^/L	22 (21)	16/213 (8)	1.93 (0.90–4.16)	0.09‡
Blood WBC 10–20 – 10^9^/L	51 (49)	109/213 (51)	- ¶	
Blood WBC ≥20 – 10^9^/L	31 (30)	88/213 (41)	0.91 (0.49–1.68)	0.76‡
Thrombocyte count – platelet/mm^3 ^$	171,000 (118,000–234,000)	214,000 (175,000–262,000)	0.67 (0.47–0.97)	0.03‡

*Table includes 320 patients in which CSF WBC was determined. Data are given as no. of patients with characteristic/no. evaluated (%) or median (interquartile range), unless indicated otherwise.

†odds ratios are calculated in 10-year increments for age, per increments of 0.2 g/L protein, per 20 mm per hour for ESR, and per 100,000/mm^3 ^for thrombocyte count.

‡significant association in univariate analysis.

§defined as the use of immuno-suppressive drugs, presence of asplenia, diabetes mellitus, alcoholism or infection with the human immunodeficiency virus.

¶this group served as a reference category.

$Glasgow Coma Scale score was obtained in 319 patients, CSF protein level was determined in 300 patients, CSF:blood glucose ratio in 294, ESR in 256, and the thrombocyte count in 295 patients.

*CSF*, cerebrospinal fluid; *WBC*, white blood-cell count; *ESR*, erythrocyte sedimentation rate.

To further explore our data we performed an analysis of correlations between indexes of inflammations in CSF and blood (Table 2). There was a significant correlation between CSF and blood WBC (Spearman's test 0.29, *P *< 0.001). CSF WBC was also significantly associated with CSF protein level (Spearman's test 0.20, *P *= 0.001), thrombocyte count (0.21, *P *< 0.001) and indexes of inflammation in the blood: erythrocyte sedimentation rate (ESR, -0.15, *P *= 0.02), and C-reactive protein level (CRP, -0.18, *P *= 0.02). Blood WBC was significantly associated with thrombocyte count (Spearman's test 0.35, *P *< 0.001) and ESR (-0.12, *P *= 0.04), but not with CRP, CSF protein or CSF glucose level.

**Table 2:** Correlations between indexes of inflammation in cerebrospinal fluid and blood

	CSF WBC	CSF glucose level	CSF protein level	Blood WBC	Thrombocyte count	ESR	CRP
CSF WBC	1.0	0.002	0.20**	0.29**	0.21**	-0.15*	-0.18*
CSF glucose level	0.002	1.0	-0.37*	-0.03	0.13*	-0.12	-0.16*
CSF protein level	0.20**	-0.37*	1.0	0.10	-0.17**	0.07	0.14
Blood WBC	0.29**	-0.03	0.10	1.0	0.35**	-0.12*	-0.06
Thrombocyte count	0.21**	0.13*	-0.17**	0.35**	1.0	0.15*	-0.18*
ESR	-0.15*	-0.12	0.07	-0.12*	0.15*	1.0	0.48**
CRP	-0.18*	-0.16*	0.14	-0.06	-0.18*	0.48**	1.0

All data are given as Spearman's correlation coefficients.

*Correlation is significant at the 0.05 level (2-tailed).

**Correlation is significant at the 0.01 level (2-tailed).

*CSF*, cerebrospinal fluid; *WBC*, white blood cell count; *ESR*, erythrocyte sedimentation rate; *CRP*, C-reactive protein.

Episodes with a CSF WBC <1000/mm^3 ^were more likely to develop systemic complications (cardiorespiratory failure and/or sepsis) during clinical course than those with higher CSF WBC (53 of 104 [51%] vs. 69 of 216 [32%]; *P *= 0.001). The proportion of episodes with neurological complications (seizures, impairment of consciousness, or focal neurological abnormalities) was similar in episodes with a CSF WBC below and above 1000 cells per mm^3 ^(84 of 104 [81%] vs. 158 of 216 [73%], respectively, *P *= 0.14).

## Discussion

Our findings indicate that a low CSF WBC in adults with pneumococcal meningitis is related to the presence of signs of sepsis and systemic complications. This is analogous to findings in patients with meningococcal meningitis [3]. Therefore, invasive pneumococcal infections should possibly also be regarded as a continuum from meningitis to sepsis, rather than of infections in isolated systemic and central nervous system compartments.

Leukocyte recruitment is a key aspect of the host response against invading micro-organisms [2]. In experimental pneumococcal meningitis blocking of leukocyte accumulation in the CSF augmented bacteraemia and increased mortality due to severe sepsis [9]. Another study in animals with pneumococcal meningitis showed a relation among a large CSF bacterial load, lack of response of CSF leukocytes and intracranial complications [10]. This suggests that low CSF WBC is related to excessive bacterial growth. In clinical studies, a low CSF WBC is a strong individual predictor of unfavourable outcome [3-5]. A recent study in experimental pneumococcal meningitis explored the role of systemic infection on meningeal inflammation [7]. In this study, meningitis was induced in rabbits by intracisternal injection of ~1 × 10^6 ^colony forming units *S. pneumoniae *type 3; WBC and bacterial concentrations were regularly determined in blood and CSF [7]. The animals were either provided with a similar dose of pneumococci intravenously at 0 hour (bacteraemic rabbits), pre-treated with paraformaldehyde-killed pneumococci (immunized rabbits) or not treated further (controls). Bacteraemic rabbits showed a decrease in blood WBC between 10–16 hours after inoculation and had an attenuated CSF pleocytosis as compared to the other groups between 12–16 hours from time of infection.

A weak leukocyte response in the CSF was significantly associated with advanced age. In a previous analysis of the Dutch Meningitis Cohort study, systemic complications have been noted as the leading causes of death in elderly patients with bacterial meningitis [11]. In the present study we lacked data for analyzing international accepted criteria of sepsis and septic shock [12]. We used a low diastolic blood pressure as a surrogate marker, which is a limitation of our study. Our results show a weak leukocyte response in the CSF was associated with bacteraemia and other factors related to systemic compromise; however, the association with a low blood WBC on admission did not reach statistical significance in our multivariate model. Therefore, a direct causal relationship between an attenuated CSF pleocytosis and a decrease in blood WBC induced by bacteraemia remains speculative.

A recent randomised, placebo-controlled trial involving 301 adults with suspected meningitis in combination with cloudy cerebrospinal fluid, bacteria in the CSF on Gram's staining, or a CSF WBC of more than 1000 per mm^3^, showed that adjunctive treatment with dexamethasone before or with the first dose of antimicrobial therapy reduced the risk of unfavourable outcome from 25 percent to 15 percent (number needed to treat, 10 patients) [13]. In this study, the effect of dexamethasone on outcome was most striking in patients with pneumococcal meningitis, in which mortality was decreased from 34 to 14 percent. A *post hoc *analysis of this study showed that the beneficial effect of dexamethasone on mortality in pneumococcal meningitis was attributable to a reduction in systemic complications, rather than neurological complications [14]. In a categorization of death of patients with pneumococcal meningitis who died within 14 days after admission, the percentage of patients who died due to neurological causes was similar between groups. The percentage of patients who died due to a systemic cause was significantly smaller in the dexamethasone group than in the placebo group (2 vs. 16%; Relative risk 0.11; 95%CI 0.04–0.25). The systemic complications that were potentially preventable by dexamethasone were pulmonary complications and septic shock [14]. As one of the inclusion criteria was a CSF WBC of more than 1000 per mm^3^, a subgroup analysis of patients included in this study with low CSF WBC on the effect of dexamethasone cannot be performed. In the current study, patients did not routinely receive corticosteroids [3]. This may well have influenced our results concerning the relation with development of complications during clinical course. However, this has not influenced our results on the correlation between CSF WBC, blood WBC and other indexes of inflammation in the blood since adjunctive dexamethasone is generally started after blood and CSF samples are obtained.

## Conclusion

We found a significant correlation between CSF WBC, blood WBC and other indexes of inflammation in the blood. In addition, a CSF WBC below 1000 per mm^3 ^was a risk factor for the development of systemic complications in patients with pneumococcal meningitis. These findings suggest that a low CSF WBC may be due the presence of bacteraemia and sepsis with early meningitis and invasive pneumococcal infections should possibly be regarded as a continuum from meningitis to sepsis.

## Competing interests

The author(s) declare that they have no competing interests.

## Authors' contributions

MW and DvdB participated in the design and coordination of the study, performed the statistical analysis, and participated in the writing of the article. JdG and LS participated in the design and coordination of the study and in the writing of the article. JBR participated in the statistical analysis and in critical revisions of the manuscript for important intellectual content. All authors read and approved the final manuscript.

## Pre-publication history

The pre-publication history for this paper can be accessed here:


